# Adipose energy stores, physical work, and the metabolic syndrome: lessons from hummingbirds

**DOI:** 10.1186/1475-2891-4-36

**Published:** 2005-12-13

**Authors:** James L Hargrove

**Affiliations:** 1 Department of Foods and Nutrition, University of Georgia, Athens, GA 30605, USA

## Abstract

Hummingbirds and other nectar-feeding, migratory birds possess unusual adaptive traits that offer important lessons concerning obesity, diabetes and the metabolic syndrome. Hummingbirds consume a high sugar diet and have fasting glucose levels that would be severely hyperglycemic in humans, yet these nectar-fed birds recover most glucose that is filtered into the urine. Hummingbirds accumulate over 40% body fat shortly before migrations in the spring and autumn. Despite hyperglycemia and seasonally elevated body fat, the birds are not known to become diabetic in the sense of developing polyuria (glucosuria), polydipsia and polyphagia. The tiny (3–4 g) Ruby-throated hummingbird has among the highest mass-specific metabolic rates known, and loses most of its stored fat in 20 h by flying up to 600 miles across the Gulf of Mexico. During the breeding season, it becomes lean and maintains an extremely accurate energy balance. In addition, hummingbirds can quickly enter torpor and reduce resting metabolic rates by 10-fold. Thus, hummingbirds are wonderful examples of the adaptive nature of fat tissue, and may offer lessons concerning prevention of metabolic syndrome in humans.

## 

Recent emphasis on human obesity obscures the fact that fat cells and the triglyceride energy system provide crucial functions in animals as diverse as invertebrate worms (*C. elegans*)[1], insects including fruit flies (*D. melanogaster*)[2], bony fish [3], toads (*Bufo *species), lizards [4], and birds [5]. Plainly, fat tissue did not originate merely as a cause of disease in sedentary people. It is part of an ancient, genetically inherited energy regulatory system in most if not all animal species. In many animals, day length and season strongly affect fat deposition through mechanisms that involve changes in pineal function, activation of the sympathetic nervous system, and changes in sensitivity to peptides such as leptin and neuropeptide Y [6,7]. Studies of seasonal weight gain offer insights into human obesity, and there may be a seasonal component in the development of human obesity in temperate regions[8].

The adaptive value of fat in providing energy for work, reproduction and survival is dramatized in the migratory energetics of the Ruby-throated hummingbird (*Archilocus colubris*), a bird which is familiar to most people who reside in eastern North America and Central America. The amount of fat (1–2 g) that would allow a human to climb about 50 feet is enough for the Ruby-throat to fly across the Gulf of Mexico, and failure to make the crossing would mean certain death. This paper will review aspects of hummingbird energetics and seasonal weight regulation that may be unfamiliar to students of human obesity.

## Biology of the Ruby-throated Hummingbird

**Figure 1:**
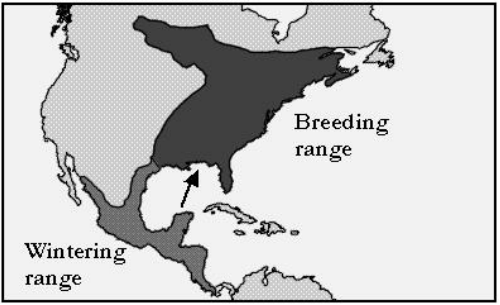
Approximate wintering range and breeding range of the Ruby-throated hummingbird. Arrow indicates a probable migratory pathway from Yucatan to the southern U.S.

The hummingbird family (Trochilidae) includes some of the smallest and most metabolically active vertebrates, with the Bumblebee hummingbird weighing under 2.0 grams [9]. At 2.5–4.8 g, the adult Ruby-throated hummingbird weighs much less than the common shrew and a little more than a U.S. penny (2.5 g). During mid-summer, females average about 3.3 g compared to 3.0 g for males [9]. Both genders contain an average of about 21% body fat (0.47–0.58 g) when not migrating [10]. The higher body weights are observed just prior to migration when the birds stop nesting and feed actively. The birds gain an additional ~1.7 g of fat and double their percent body fat prior to migration [11]. *A. colubris *spends the winter in Central America and migrates to North America for the breeding season, going as far north as Ontario, Canada (Fig. 1). The total trip may exceed 2000 miles, and is reversed in the fall. The migrations are timed to coincide with the blossoming of several flowering plants, which provide nectar that fuels much of the journey. Nectars contain as much as 38% (~1 M) sugars (mostly sucrose) [12,13]. After fasting overnight, hummingbirds primarily metabolize free fatty acids and have a respiratory quotient (RQ) of about 0.7. However, the RQ quickly goes to about 1.0 when they begin to feed, indicating oxidation of carbohydrate [14,15]. During the breeding season, males maintain an extremely accurate body mass by ingesting small meals roughly every 15–20 min, and using the energy to court females and chase away other males [16]. Just before nightfall, they consume enough nectar to last the night; when that fails, they may enter torpor to conserve energy [15,17].

Hummingbirds have one of the highest metabolic rates relative to metabolic body size of any animal on earth. Heart rates up to 1260 beats per minute have been recorded, and breathing rate is about 250 breaths per minute even at rest. Resting body temperatures are about 39°C [9]. At rest, oxygen consumption is about 4 ml O_2_/g/h [18]. During flight, hummingbird oxygen consumption per gram of muscle tissue is approximately 10 times higher than that seen for elite human athletes [19].

Preparation for migration requires that the birds switch from carbohydrate to fat metabolism during flight, and this entails changes in feeding behavior, energy storage and mitochondrial energy usage. During periods of rapid fattening, hummingbird RQ values are above 1.0, consistent with lipogenesis or at least fat storage. The mechanisms of fattening include increased energy intake, increased food efficiency, altered diet selection, and increased lipogenic enzymes [20]. Preferential metabolism of carbohydrate spares lipids for storage. Hummingbirds do eat insects and they may increase insect consumption prior to migration [21,22].

Hummingbirds [13,23] and many other birds [5,24] maintain very high blood glucose both in the fasted and fed conditions. In hummingbirds, fasted glucose is about 17 mM (300 mg/dl), and it increases to about 42 mM (740 mg/dl) after feeding [23,25]. Although these levels would be classified as diabetic in humans, nectivorous birds do not become diabetic [13,26] in the traditional sense of spilling glucose into the urine with symptoms of polyuria, polydipsia and polyphagia. Also, they do not develop the degree of glycated hemoglobin seen in humans [23]. Migrating birds may become insulin resistant and there may be a parallel to human metabolic syndrome [27]. However, the birds are forced to switch from almost total reliance on carbohydrate to almost total reliance on fatty acid metabolism during migration over oceans or desert terrain that provides no other energy sources.

## Hummingbird Energetics: Across the Gulf of Mexico on a Gram of Fat

**Figure 2:**
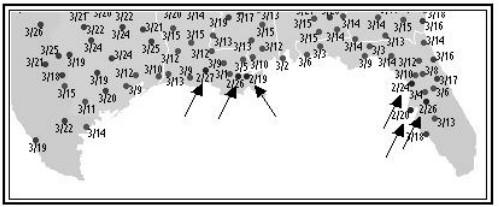
First Spring sightings of Ruby-throated hummingbirds in 2005 occurred during February in Louisiana and Florida (arrows) before the birds had been reported in Texas. Source: Hummingbirds.net .

During the northward migration, many ruby-throated hummingbirds reach the Gulf of Mexico on the coast of Yucatan. The distance to the U.S. can equal 500–600 miles, and the most direct routes provide no sites at which food or water may be obtained. Whereas some birds may take a coastal route or possibly fly to Cuba, ornithologists believe that most birds fly non-stop across the Gulf of Mexico [9]. Networks of bird-watchers report data on various species during the migrations, and it is noteworthy that arrivals may be reported in Louisiana and Florida prior to arrivals in Texas (Fig. 2). Males arrive in the U.S. prior to the females, and time is critical because the birds compete for habitat. A premium may be placed on early arrival because it provides selective advantage in breeding. Prior to departing, the birds must store enough energy to fly at speeds that range from 25–50 mph.

Pearson [28] measured oxygen consumption of 2 species of hummingbird during hovering flight and found values of 68–85 ml O_2_/g/hr. He calculated a flight range of 385 miles on the assumption that the birds stored 1 g of fat and consumed 80 ml O_2_/g/hr (caloric equivalent of 4.69 kcal/l) when flying at 50 mph. However, Odum et al [11] showed that Ruby-throated hummingbirds can store more than 40% of body weight as fat, and found a mean content of fat of 2.25 g in birds accidentally killed at television towers. Lasiewski [29] showed that the metabolic rate is probably lower (42 cc.O_2_/gm/hr) than Pearson estimated. Assuming a flight speed of 25 mph, he estimated that males have a flight range of about 650 miles while females have a range of about 610 miles. The highest estimate of flight range for the Ruby-throated hummingbird is about 2500 km (1500 miles) [11].

If one assumes an average flight weight of 3.5 g and a caloric equivalent of 4.69 kcal/l, then the energy needed for crossing the Gulf of Mexico is about 0.7 kcal per hour. A 20 hour crossing would require about 14 kcal, or 1.5 g of fat (1.8 g of fat tissue assuming that fat tissue contains 7.7 kcal/g). If the birds were to rely on glycogen for this energy, they would need to store about 3.5 g of carbohydrate. Adding 2 g of water of hydration for each g of glycogen, the birds would have to increase their body weight to above 10 g! The flight is possible only because the energy yield per gram of fat is 10 fold higher than for hydrated glycogen [19]. If they relied on glycogen, it is unlikely that the birds could generate sufficient lift to leave Yucatan, much less carry the extra weight across the Gulf of Mexico [22].

## Conclusion

Migrating birds stay lean until pre-migratory fattening becomes necessary, and then may add fat at a rate of 1–13% of body weight per day [27]. For Ruby-throated hummingbirds, the fattening is crucial for survival. The average "field metabolic rate" is about 8 times resting metabolic rate [30], and during constant flight, hummingbirds expend about 0.7 kcal/hr [18,29]. Glucose at 42 mM distributed in blood and extracellular fluid (20% of body weight) would provide 6 mg of glucose (24 calories or 100 J). This is sufficient only for a few minutes of flight. The birds would quickly shift to metabolism of glycogen and fatty acids to provide adequate energy. Thus, the pre-migratory fattening is purposive, and a typical flight across the Gulf of Mexico will require about 75% of the birds' energy stores (assuming that 1.5 g of fat is used out of ~2.0 g stored).

The first lesson that the Ruby-throated hummingbird teaches is that becoming fat can be beneficial if it is necessary as an energy buffer to survive. The second lesson is that fat birds with very high plasma glucose levels do not become diabetic. Part of the preventative mechanism is anatomical and physiological. Nectar feeding birds are unusual in that they consume large amounts of water along with the sugars they typically consume [26]. Birds have a relatively low glomerular filtration rate and are able to reabsorb essentially all of the glucose that is filtered into the urine [26]. It is not clear how they avoid showing symptoms of "glucose toxicity" such as glycated hemoglobin, but the levels of hemoglobin A1c are lower than in humans [23]. One hypothesis could be that the turnover rates of red blood cells and proteins are substantially higher in birds than in mammals. For example, the lifespan of red blood cells in birds can be 21 days or less vs. about 120 days for humans[31], so there may be less opportunity for glycation. Turnover rates for metabolic pools are thought to be proportional to body mass to the 1/4 power [32], which would indicate that metabolic pools exchange about 12 times faster in hummingbirds than in humans (Kleiber, p. 216 and 390). This is congruent with the high ATP turnover rate in active muscle [33]. Whether or not birds avoid obesity and diabetes by dint of their rates of living, the neurobiology and endocrinology of avian fat deposition are complex, and students of migratory birds have suggested that they could offer important clues concerning prevention of obesity and diabetes in humans [26,27].

Diabetes and the metabolic syndrome are rightfully considered to be kinetic disorders that do not develop unless several major controls fail. Typically, sensitivity to insulin and to glucose ("glucose effectiveness") both diminish, creating insulin resistance [34]. Hepatic glucose production may continue (instead of shutting off) even if plasma glucose and insulin are both elevated. The rate of production of insulin by the beta cell must also fail to compensate for the decreased sensitivity [34]. These are conditions that come about in humans because of sedentary habits and obesity [35]. In migrating birds, there may be a decline in insulin sensitivity, but it is unlikely that regulation of beta cell function or hepatic glucose production becomes abnormal. Hummingbirds combat kinetic disorders by dint of their highly aerobic lifestyles and necessity of maintaining close feedback between energy intake and energy expenditure. Unlike humans who have "uncoupled" food intake from functional needs, animals that must flap their wings at 50 beats per second in order to feed have a hard time staying fat.

## Competing interests

The author(s) declare that they have no competing interests.

## Authors' contributions

This review was prepared entirely by Dr. Hargrove.
